# Development of Growth Factor Releasing Hyaluronic Acid-Based Hydrogel for Pulp Regeneration: A Preliminary Study

**DOI:** 10.3390/gels8120825

**Published:** 2022-12-13

**Authors:** Mi Sun Kim, Yu-Shik Hwang, Hyo-Seol Lee, Ok Hyung Nam, Sung Chul Choi

**Affiliations:** 1 Department of Pediatric Dentistry, Kyung Hee University College of Dentistry, Kyung Hee University Hospital at Gangdong, 892 Dongnam-ro, Gangdong-gu, Seoul 05278, Republic of Korea; 2 Department of Maxillofacial Biomedical Engineering, Kyung Hee University College of Dentistry, 26 Kyungheedae-ro, Dongdaemoon-gu, Seoul 02447, Republic of Korea; 3 Department of Pediatric Dentistry, Kyung Hee University College of Dentistry, Kyung Hee University Medical Center, 26 Kyungheedae-ro, Dongdaemoon-gu, Seoul 02447, Republic of Korea

**Keywords:** fibroblast growth factor-2, growth factor, hyaluronic acid-collagen hybrid hydrogel, platelet-derived growth factor-BB, pulp regeneration

## Abstract

Growth factors play essential roles as signaling molecules in pulp regeneration. We investigated the effect of a hyaluronic acid (HA)-collagen hybrid hydrogel with controlled release of fibroblast growth factor (FGF)-2 and platelet-derived growth factor (PDGF)-BB on human pulp regeneration. The cell interaction and cytotoxicity of the HA-collagen hybrid hydrogel, the release kinetics of each growth factor, and the effects of the released growth factors on pulp cell proliferation were examined. The vitality of pulp cells was maintained. The amounts of FGF-2 and PDGF-BB released over 7 days were 68% and 50%, respectively. Groups with a different concentration of growth factor (FGF-2: 100, 200, 500, and 1000 ng/mL; PDGF-BB: 10, 50, 100, 200, and 500 ng/mL) were experimented on days 1, 3, 5, and 7. Considering FGF-2 concentration, significantly increased pulp cell proliferation was observed on days 1, 3, 5, and 7 in the 100 ng/mL group and on days 3, 5, and 7 in the 200 ng/mL group. In the case of PDGF-BB concentration, significantly increased pulp cell proliferation was observed at all four time points in the 100 ng/mL group and on days 3, 5, and 7 in the 50, 200, and 500 ng/mL groups. This indicates that the optimal concentration of FGF-2 and PDGF-BB for pulp cell proliferation was 100 ng/mL and that the HA-collagen hybrid hydrogel has potential as a controlled release delivery system for FGF-2 and PDGF-BB.

## 1. Introduction

The traditional root canal therapy technique involves devitalization of a tooth, which predisposes it to reinfection and fracture. Recently, regenerative endodontics has gained much attention as a promising biologically based alternative treatment. The ultimate goal of pulp regeneration is to reconstitute the vitality and function of the dental pulp due to trauma or infection [1]. For ingrowth of new regenerated tissues, advances in tissue engineering research have focused on three key elements for regeneration: stem cells, which have the ability to proliferate and differentiate; scaffold, which is a three-dimensional structure that supports the integrity of the tissue; and growth factors, which are signals governing morphogenesis and differentiation [2,3].

**Figure 1:**
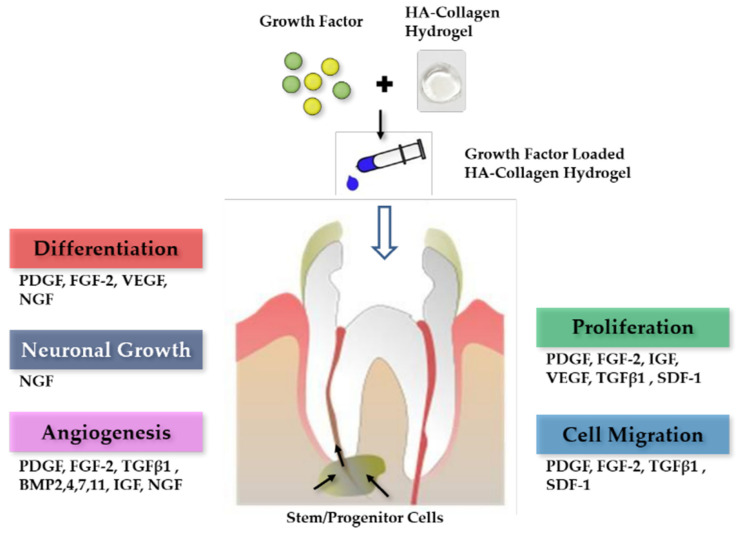
Signaling molecules for pulp regeneration. PDGF, platelet-derived growth factor; FGF, fibroblast growth factor; VEGF, vascular endothelial growth factor; NGF, nerve growth factor; TGF, transforming growth factor; BMP, bone morphogenetic protein; IGF, insulin-like growth factor; SDF, stromal cell-derived factor [1].

Growth factors are secreted polypeptides or proteins that control various cellular responses, such as cell migration, proliferation, differentiation, angiogenesis, neural growth, and apoptosis [1] (Figure 1). Growth factors act as signaling molecules in cellular activities by binding to receptors present on the cell membranes of target cells [4]. After injury to dental pulp tissue, various growth factors participate in tissue repair. Therefore, growth factors are being increasingly considered to induce pulp regeneration.

Fibroblast growth factor (FGF)-2 is a multifunctional heparin-binding protein that stimulates and modulates cell growth, differentiation and migration [5]. Among the 22 FGF types identified in humans, FGF-2 was recently demonstrated to stimulate the regenerative potential of the dentin-pulp complex, increase proliferation and migration and regulate the cytodifferentiation of odontoblasts [6,7] (Table 1). Platelet-derived growth factor (PDGF) exerts a growth-promoting activity in human platelet α-granules and is a 30-kDa dimeric molecule with disulfide bonded A- and B-polypeptide chains [8,9]. PDGF has homodimeric (PDGF-AA, PDGF-BB, PDGF-CC, and PDGF-DD) and heterodimeric (PDGF-AB) isoforms [10]. It is a potent chemotactic, mitogenic, and activating agent for cells that are essential in soft tissue repair [11,12,13]. PDGF also stimulates the proliferation of fibroblasts in human dental pulp and angiogenesis at the site of dental pulp injury [14,15] (Table 1). Hence, although several growth factors appear to be involved in pulp regeneration, two key growth factors (FGF and PDGF) were investigated in this study.

**Figure 2:**
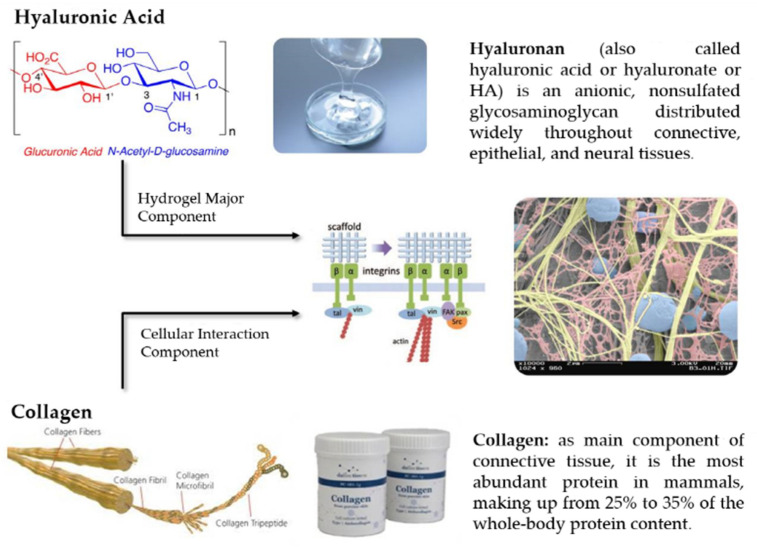
Hyaluronic acid and collagen are two of the most extensively studied and frequently used forms of naturally derived biomaterials in tissue engineering.

A suitable delivery system is essential for sustained release of the growth factors with progression of dentine dissolution in caries lesions or from the injured pulp cells. Because growth factors could be denatured easily in aqueous solutions at 37 °C, their biological activity should be preserved for effective application over extended periods [16]. Therefore, bioactive molecule delivery systems have been extensively investigated. To date, numerous synthetic and naturally derived polymers have been used as depots and delivery vehicles for protein growth factors [24]. Among them, hydrogels are one of the most extensively studied and frequently used forms of naturally derived biomaterials in tissue engineering [25]. The development of scaffolds from biomaterials, such as hyaluronic acid (HA) and collagen with improved mechanical stability against degradation and with better biochemical function can help deliver growth factors to promote regeneration [26] (Figure 2). The physiological activities of HA and collagen have a virtue in various applications in tissue engineering [27].

**Figure 3:**
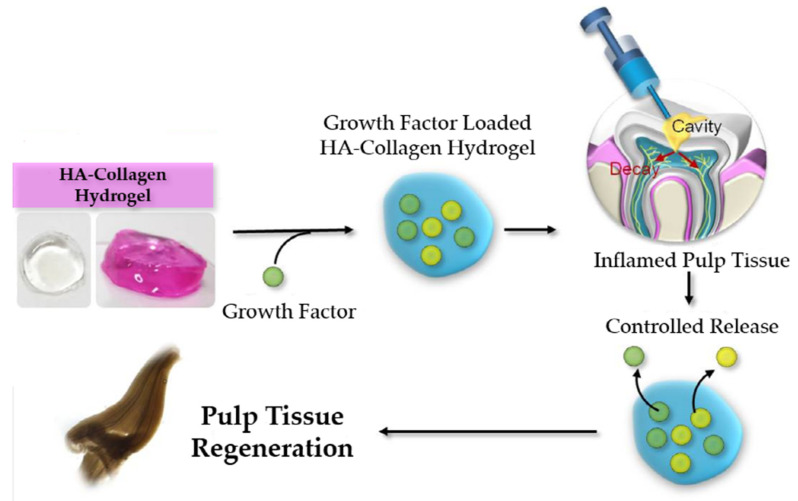
A schematic description of the use of growth factors released from HA-collagen hydrogel for pulp tissue regeneration.

Based on these findings, FGF-2 and PDGF-BB loaded HA-collagen hybrid hydrogels were developed as a sustained release delivery system to maintain the biological activity of growth factors. The hypothesis of this study was that the effective proliferation of the human pulp cells can be achieved by using growth factor loaded HA-collagen hybrid hydrogels. To test this hypothesis, we investigated the effect of these HA-collagen hybrid hydrogels with controlled release of FGF-2 and PDGF-BB on human pulp cells (Figure 3).

## 2. Results and Discussion

### 2.1. Characterization of the HA + Collagen Hybrid Hydrogel 

**Figure 4:**
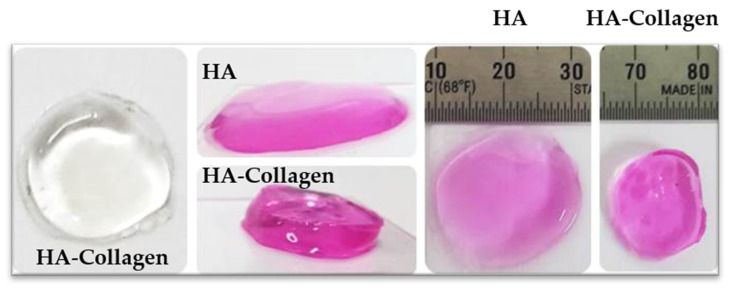
Optical images of HA-collagen hybrid gel and HA hydrogel. The mechanical integrity of HA-collagen hydrogel was increased compared with HA hydrogel. HA, hyaluronic acid.

**Figure 5:**
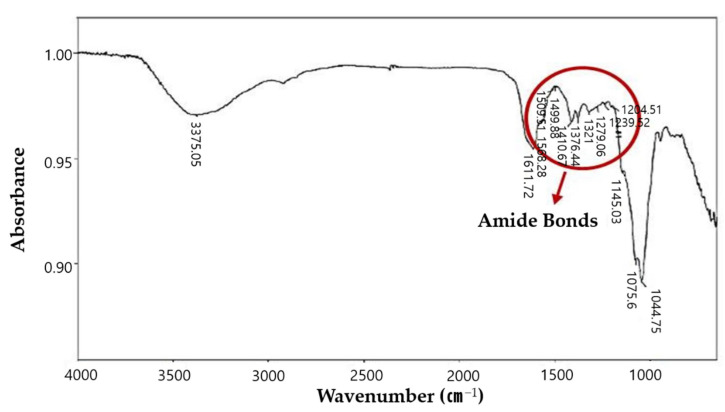
Fourier transform infrared spectroscopy. Spectrum of HA-collagen hydrogel. Infrared spectrometry showed the presence of amide bonds which are the reaction outcome of adipic dihydrazide (ADH) and oxidized HA. HA, hyaluronic acid.

The mechanical integrity of the HA-collagen hydrogel was higher than that of the HA hydrogel alone (Figure 4). Infrared spectrometry was performed to determine the chemical modifications to the HA during the crosslinking reaction (Figure 5). The presence of amide bonds, which are the result of adipic dihydrazide (ADH) and oxidized HA reactions, was observed.

### 2.2. Release Kinetics

**Figure 6:**
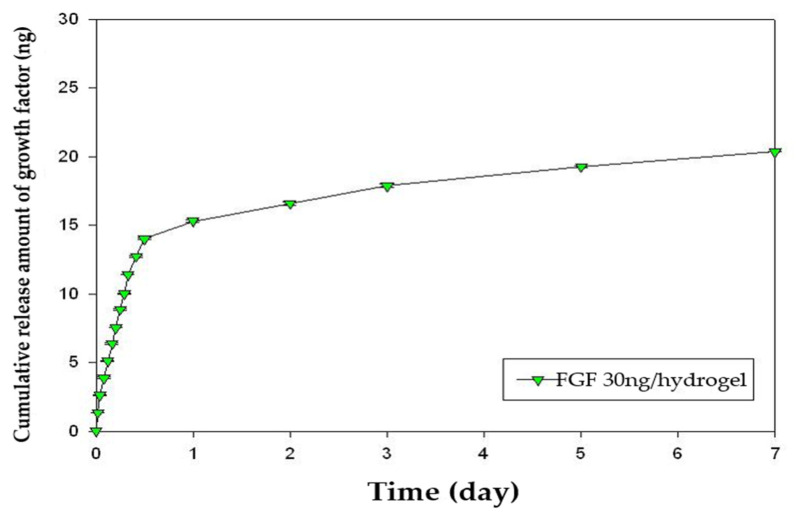
Cumulative release profile of FGF-2 over 7 days. The release kinetics of growth factors was quantified by an enzyme-linked immunosorbent assay (ELISA). FGF-2 was released sustainably for 7 days but significantly during the first 24 h. The released amount of FGF-2 for 7 days was 68%. FGF, fibroblast growth factor.

**Figure 7:**
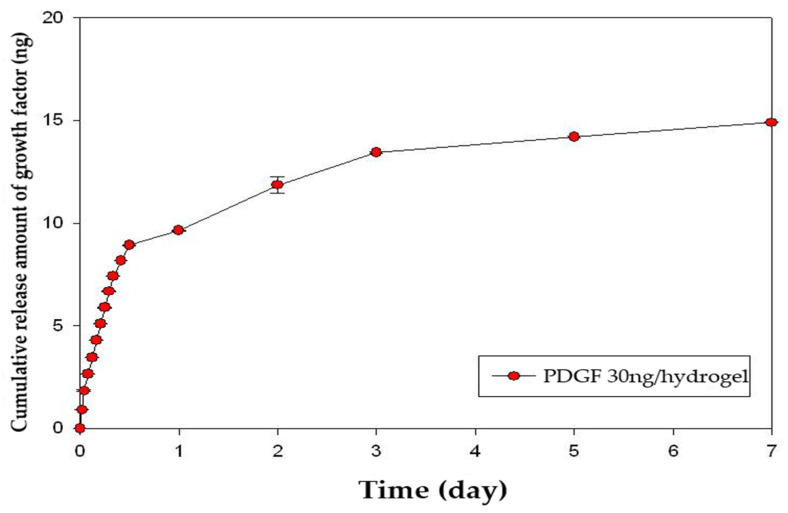
Cumulative release profile of PDGF-BB over 7 days. The release kinetics of growth factors was quantified by an enzyme-linked immunosorbent assay (ELISA). PDGF-BB was released sustainably for 7 days but significantly during the first 24 h. The released amount of PDGF-BB for 7 days was 50%. PDGF, platelet-derived growth factor.

The release kinetics of growth factors were quantified using an enzyme-linked immunosorbent assay (ELISA). The amount of FGF-2 released over 7 days was 68% (Figure 6). The amount of PDGF-BB released over 7 days was 50% (Figure 7). Sustained release of both FGF-2 and PDGF-BB was observed for 7 days, but significant release was observed during the first 24 h. 

### 2.3. Cellular Interaction and Cytotoxicity Test 

**Figure 8:**
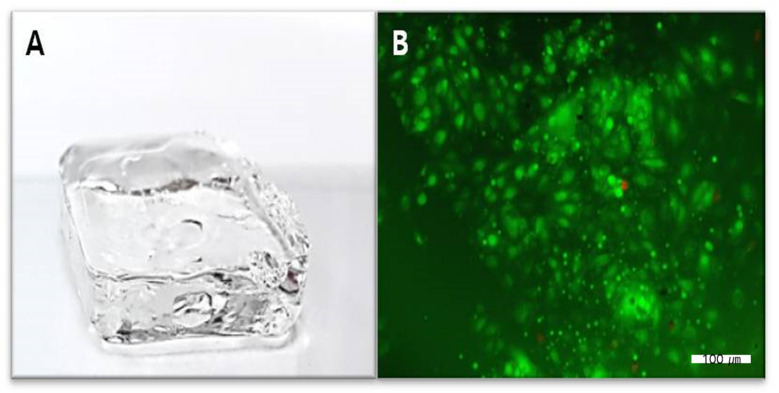
(**A**) Pulp cells in HA-collagen hydrogel (**B**) Live and dead analysis image shows that dental pulp cells adhered well to the HA-collagen hydrogel, proliferated progressively, and maintained cell viability with green fluorescence. HA, hyaluronic acid.

**Figure 9:**
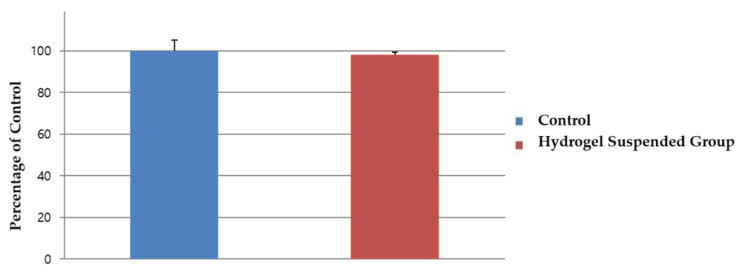
Findings of the cell viability test performed using a cell counting kit (CCK)-8 kit. The cell viability was not affected by the presence of the hydrogel.

The live and dead analysis, (Figure 8) revealed that dental pulp cells adhered well to the HA-collagen hydrogel, proliferated progressively, and maintained cell viability. Moreover, HA-collagen hydrogels did not exert significant cytotoxicity (Figure 9).

### 2.4. Effect of Growth Factors on Cell Proliferation

**Figure 10:**
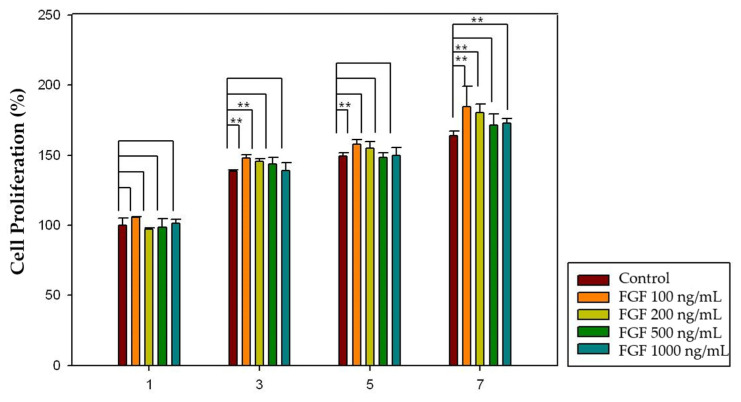
The effect of FGF-2 on pulp cell proliferation. The 100, 200 and 1000 ng/mL concentrations showed significantly increased pulp cell proliferation rates in the 3-, 5-, and 7-day groups (*p* < 0.05). The 100 ng/mL concentration showed significantly increased pulp cell proliferation rates in all four groups (*p* < 0.05). The results are expressed as mean values ± standard deviation. ** indicates the statistically significant differences (*p* < 0.05). FGF, fibroblast growth factor.

**Figure 11:**
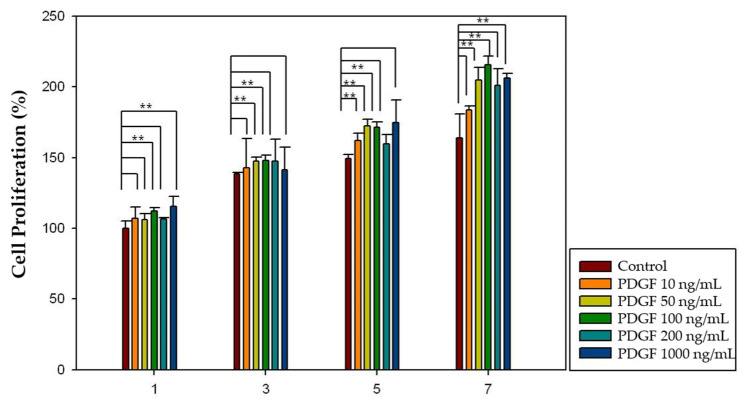
The effect of PDGF-BB on pulp cell proliferation. The 50, 100, and 500 ng/mL concentrations showed significantly increased pulp cell proliferation rates in the 3-, 5-, and 7-day groups (*p* < 0.05). The 100 ng/mL concentration showed significantly increased pulp cell proliferation rates in all four groups (*p* < 0.05). The results are expressed as mean values ± standard deviation. ** indicates the statistically significant differences (*p* < 0.05). PDGF, platelet-derived growth factor.

Different concentrations of growth factor (FGF-2: 100, 200, 500, and 1000 ng/mL; PDGF-BB: 10, 50, 100, 200, and 500 ng/mL) and increased incubation time (1, 3, 5, and 7 days) were applied to investigate the effect of growth factors on dental pulp cell proliferation. Considering FGF-2, 100, 200 and 1000 ng/mL concentrations showed significantly increased pulp cell proliferation rates in the 3-, 5-, and 7-day groups (*p* < 0.05), and 100 ng/mL concentration showed significantly increased pulp cell proliferation rates in all four groups (*p* < 0.05) (Figure 10). Moreover, in the case of PDGF-BB, 50, 100 and 500 ng/mL concentrations showed significantly increased pulp cell proliferation rates in the 3-, 5-, and 7-day groups (*p* < 0.05), and 100 ng/mL concentration showed significantly increased pulp cell proliferation rates in all four groups (*p* < 0.05) (Figure 11). The proliferation rate of both growth factors increased as the incubation time increased.

### 2.5. Effect of Growth Factors Released from HA-Collagen Hydrogel on Cell Proliferation

**Figure 12:**
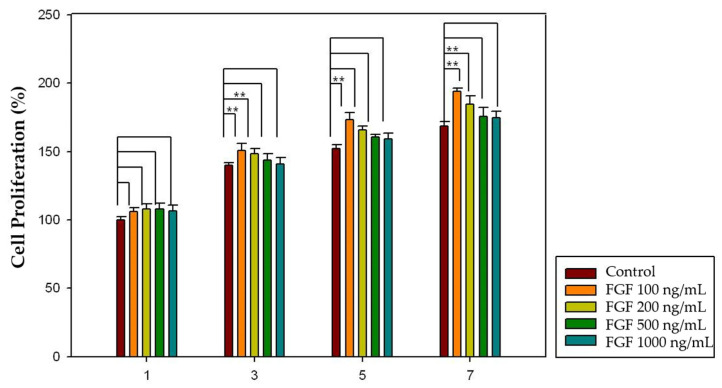
The effect of FGF-2 released from HA-collagen hydrogel on pulp cell proliferation. The 100 and 200 ng/mL concentrations showed significantly increased pulp cell proliferation rates in the 3-, 5-, and 7-day groups (*p* < 0.05). The 100 ng/mL concentration showed significantly increased pulp cell proliferation rates in all four groups (*p* < 0.05). The results are expressed as mean values ± standard deviation. ** indicates the statistically significant differences (*p* < 0.05). FGF, fibroblast growth factor.

**Figure 13:**
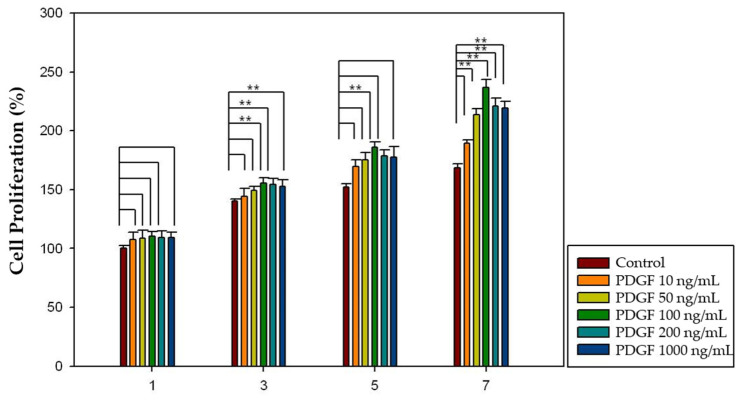
The effect of PDGF-BB released from HA-collagen hydrogel on pulp cell proliferation. The 50, 100, 200 and 500 ng/mL concentrations showed significantly increased pulp cell proliferation rates in the 3-, 5-, and 7-day groups (*p* < 0.05). The 100 ng/mL concentration showed significantly increased pulp cell proliferation rates in all four groups (*p* < 0.05). The results are expressed as mean values ± standard deviation. ** indicates the statistically significant differences (*p* < 0.05). PDGF, platelet-derived growth factor.

To determine the effect of growth factors released from the HA-collagen hydrogel on dental pulp cell proliferation, different concentrations of growth factor (FGF-2: 100, 200, 500, and 1000 ng/mL; PDGF-BB: 10, 50, 100, 200, and 500 ng/mL) and increased incubation time (1, 3, 5, and 7 days) were applied. In the case of FGF-2, 100 and 200 ng/mL concentrations showed significantly increased pulp cell proliferation rates in the 3-, 5-, and 7-day groups (*p* < 0.05), and 100 ng/mL concentration showed significantly increased pulp cell proliferation rates in all four groups (*p* < 0.05) (Figure 12). Moreover, in the case of PDGF-BB, 50, 100, 200 and 500 ng/mL concentrations showed significantly increased pulp cell proliferation rates in the 3-, 5-, and 7-day groups (*p* < 0.05) and 100 ng/mL concentration showed significantly increased pulp cell proliferation rates in all four groups (*p* < 0.05) (Figure 13). The proliferation rates of both growth factors increased as incubation time increased.

### 2.6. Discussion

To date, studies on dental pulp regeneration have been carried out by the typical tissue engineering approaches of delivering cells in biomaterial scaffolds with tooth slices or fragments for in vivo implantation [3,28,29]. Recently, an in vitro level cell-homing approach has been used to regenerate the pulp cells by the delivering growth factors [30]. Several growth factors, including FGF-2, vascular endothelial growth factor, nerve growth factor, PDGF, and bone morphogenetic protein-7, have been applied singularly or in combination into the root canal spaces. Dental pulp-like tissues can be regenerated with new blood vessels, indicating that these tissues can be regenerated without cell implantation [30]. This approach to dental pulp regeneration by applying multiple growth factors may accelerate the clinical translation of these findings.

Numerous growth factor delivery systems for the cell-homing approach have been investigated over the past decade. New approaches have been developed based on the molecular and cellular signaling pathways that regulate the regeneration process and their potential for clinical application [31]. In particular, hydrogels have numerous applications in tissue engineering and drug delivery. Hydrogels are structurally similar to the molecular components in the body and are considered biocompatible [32]. Moreover, hydrogels can be injected into the target sites. This approach enables clinicians to use hydrogels in a minimally invasive manner.

In tissue engineering, hydrogels have a number of design criteria to function appropriately and promote new tissue formation [33]. HA is a glycosaminoglycan component of natural extracellular matrices and is known to play a significant role in wound healing. However, HA hydrogels typically have poor mechanical properties and low stability against enzymatic degradation, which makes HA more suitable as an additive to another polymer such as collagen [34]. Therefore, HA-collagen hybrid hydrogels were prepared in this study. To investigate the cytotoxicity of the HA-collagen hybrid hydrogel with dental pulp cells, CCK-8 and live and dead assays were performed to ensure that they were not toxic. Our results showed that HA-collagen hydrogels can be used as a nontoxic delivery system for growth factors.

The release kinetics of FGF-2 and PDGF-BB showed that the HA-collagen hybrid hydrogel could be used as a controlled-release delivery system for FGF-2 and PDGF-BB in the early steps of pulp regeneration. We observed sustained release for 7 days, and the biological effects of FGF-2 and PDGF-BB on pulp cell proliferation were maintained. Furthermore, there was a significant release during the first 24 h, which may be attributed to a burst effect [35]. The release rate of FGF-2 was greater than that of PDGF-BB. This difference in the release rates can be caused by the different molecular nature and affinity of these growth factors to the polymer, such as molecular weight, hydrophobicity and electro charge differences.

A cell proliferation assay using the CCK-8 kit was performed to investigate the effect of growth factors on pulp cell proliferation. The proliferation of dental pulp cells increased as the incubation time increased. Moreover, there was an optimal concentration of each growth factor that affected the proliferation of dental pulp cells. In the case of FGF-2, 100, 200, and 1000 ng/mL concentrations showed significantly increased pulp cell proliferation rates in the 3-, 5-, and 7-day groups (*p* < 0.05) and 100 ng/mL concentration showed significantly increased pulp cell proliferation rates in all four groups (*p* < 0.05) (Figure 12). Moreover, in the case of PDGF-BB, 50, 100, and 500 ng/mL concentrations showed significantly increased pulp cell proliferation rate in the 3-, 5-, and 7-day groups (*p* < 0.05) and 100 ng/mL concentration showed significantly increased pulp cell proliferation rates in all four groups (*p* < 0.05) (Figure 13). 

**Figure 14:**
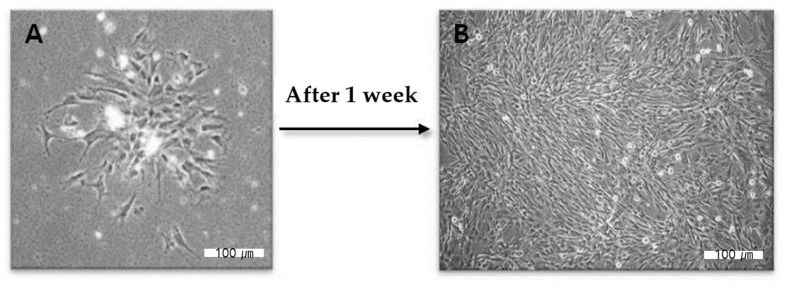
(**A**,**B**) Proliferation of human dental pulp cells over a week. Figures were taken from an optical microscope.

**Figure 15:**
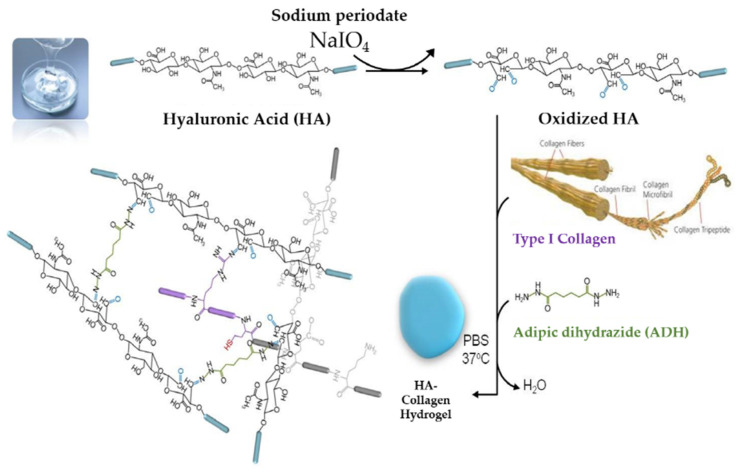
The synthetic scheme of hyaluronic acid (HA)-collagen hybrid hydrogel preparation. Oxidized HA was first formed by reacting sodium periodate and HA. Because adipic dihydrazide (ADH) works as a cross-linking agent, the hybrid-hydrogel was cured and hardened by mixing oxidized HA, collagen and ADH. Finally, the hydrogel with HA and collagen backbone at 37 °C was developed. Blue rod indicates the polymer of HA; purple rod indicates the polymer of collagen; grey rod indicates the polymer of ADH.

Finally, a cell proliferation assay using the CCK-8 kit was performed to investigate the effect of the growth factors released from the HA-collagen hydrogel on pulp cell proliferation. The proliferation of dental pulp cells increased as the incubation time increased. Moreover, there was an optimal concentration of each growth factor that affected the proliferation of dental pulp cells. The results seem to be quite close to those of the growth factor only groups. In the case of FGF-2, 100 and 200 ng/mL concentrations showed significantly increased pulp cell proliferation rates in the 3-, 5-, and 7-day groups (*p* < 0.05) and 100 ng/mL concentration showed significantly increased pulp cell proliferation rates in all four groups (*p* < 0.05) (Figure 14). Moreover, in the case of PDGF-BB, 50, 100, 200 and 500 ng/mL concentrations showed a significantly increased pulp cell proliferation rate in the 3-, 5-, and 7-day groups (*p* < 0.05) and 100 ng/mL concentration showed significantly increased pulp cell proliferation rates in all four groups (*p* < 0.05) (Figure 15). The proliferation rates of both growth factors increased as the incubation time increased. 

Because the price of growth factors is relatively high, it is important to determine the optimal concentration of growth factors to develop a clinically applicable delivery system for regenerative endodontics. A low concentration of growth factors showed less effect due to the inadequate amount, whereas a high concentration suppressed the proliferation because the excess of the growth factor might not participate in growth factor-receptor signaling responses. Although the optimal concentration of growth factors has been found within the limits of this study, further evaluation of the subsequent effects on the pulp regeneration processes is needed. The limitation that hydrogel itself could act as a barrier or even an obstacle to pulp regeneration should also be considered. In this regard, it is important to investigate and use the optimal amounts of hydrogel to avoid these side effects. Moreover, many different kinds of hydrogels with various mechanical characteristics can be developed. Therefore, it is important to develop the most suitable hydrogel with the proper mechanical characteristics in need. 

## 3. Conclusions

Although the findings of previous studies on pulp regeneration are very favorable, biologically and clinically complete pulp regeneration has not yet been achieved. The present study demonstrated that the HA-collagen hybrid hydrogel can be used as a controlled-release delivery system for FGF-2 and PDGF-BB. Moreover, within the limits of this study, the optimal concentration of both FGF-2 and PDGF-BB for the proliferation of dental pulp cells was found to be 100 ng/mL. Further research should focus on other ranges of cellular activities in pulp regeneration that growth factors can affect, including migration, differentiation, and apoptosis of all dental pulp cells and other growth factors involved in pulp regeneration. 

## 4. Materials and Methods

### 4.1. Human Pulp Cells Preparation

Human dental pulp cells were purchased from Lonza Inc. (Walkersville, MD, USA) and cultured in 100-mm-diameter culture dishes (SPL Life Sciences, Seoul, Republic of Korea) containing alpha minimum essential medium (Gibco BRL, Eggenstein, Germany) supplemented with 10% fetal bovine serum (Gibco BRL Eggenstein, Germany), 1% penicillin-streptomycin (Gibco BRL Eggenstein, Germany), 100 μM ascorbic acid (Sigma aldrich, St. Louis, MO, USA), and 200 mM L-glutamine (Gibco BRL Eggenstein, Germany) in a humidified atmosphere containing 5% CO_2_ at 37 °C. Confluent cultures were collected by trypsinization and subcultured in 100-mm-diameter culture dishes (SPL Life Sciences, Seoul, Republic of Korea) (Figure 14).

### 4.2. HA + Collagen Hybrid-Hydrogel Preparation and Characterization

To develop HA and collagen hybrid-hydrogel with optimal mechanical properties, a pilot study on the proportion of HA to collagen was performed and the adequate ratio of HA to collagen was 4:1. Oxidized HA was first formed by the reaction of sodium periodate and HA. Because adipic dyhydrazide (ADH) works as a cross-linking agent, the hybrid-hydrogel was cured and hardened by mixing oxidized HA, collagen and ADH. Finally, a hydrogel with HA and a collagen backbone at 37 °C was developed (Figure 15). The developed hybrid-hydrogel was characterized using Fourier transform infrared spectroscopy (FT-IR). 

### 4.3. Release Kinetics Study

To control the release of growth factors, the kinetics of HA-collagen hydrogels containing growth factors (30 ng/hydrogel) were dispersed into test tubes containing 3 mL Dulbecco’s phosphate-buffered saline (DPBS) solution (Gibco BRL, Eggenstein, Germany). The suspension was gently stirred at room temperature in a release chamber. DPBS solutions in the tubes were collected 16 times at specific time points for 7 days and analyzed for growth factor secretion through an enzyme-linked immunosorbent assay (ELISA) in accordance with the manufacturer’s instruction (Peprotech, Rocky Hill, NJ, USA). The experiments were conducted at least three times by one skilled operator. 

### 4.4. Cellular Interaction and Cytotoxicity Test 

To evaluate the cellular interaction of the HA-collagen hybrid hydrogel, a live and dead assay was performed. To check the toxicity of the hydrogel, a cell counting kit-8 (CCK-8; Dojindo Lab., Kumamoto, Japan) was used. Dental pulp cells were plated at 5000 cells/well in 96-well plates (SPL Life Sciences, Seoul, Republic of Korea). The 96-well dishes were then placed in a humidified incubator containing 5% CO_2_ at 37 °C for 24 h before use. After 24 h, the medium from each well was removed and replaced with 100 µL of test or control medium. The hydrogel was suspended in 1 mL alpha-MEM culture medium without bovine serum for 24 h. The same medium without contact with the hydrogel was used as the control. The 96-well dishes were placed in a humidified incubator containing 5% CO_2_ at 37 °C for 48 h. The CCK-8 solution was diluted to 1/10 with alpha-MEM without bovine serum. The medium was removed and replaced with 100 µL/well diluted CCK-8 solution. The 96-well dishes were placed in a humidified incubator containing 5% CO_2_ at 37 °C for 2 h. The absorbance of each 96-well dish was determined using an automatic microplate spectrophotometer (Bio-Rad, Hercules, CA, USA) at 450 nm (OD 450). The experiments were conducted at least three times by one skilled operator. 

### 4.5. Effect of Growth Factors on Cell Proliferation

Different concentrations of growth factors were suspended in alpha-MEM without bovine serum. (FGF-2: 100, 200, 500, and 1000 ng/mL; PDGF-BB: 10, 50, 100, 200, and 500 ng/mL). For the proliferation assay, the CCK-8 kit was used as described previously on days 1, 3, 5, and 7. Control groups without growth factors were used (100% viability). The results were normalized and expressed as a percentage of the optical density values of the control groups. The experiments were conducted at least three times by one skilled operator. 

### 4.6. Effect of Growth Factors Released from HA-Collagen Hydrogel on Cell Proliferation

Different concentrations of growth factors loaded in the HA-collagen hybrid hydrogel (FGF-2: 100, 200, 500, and 1000 ng/mL; PDGF-BB: 10, 50, 100, 200, and 500 ng/mL) were placed in cell culture inserts (SPL Life Sciences), and the released growth factors were diffused into alpha-MEM culture medium without bovine serum. For the proliferation assay, the CCK-8 kit was used as described previously on days 1, 3, 5, and 7. Unloaded hydrogels were used as the controls (100% viability). The results were normalized and expressed as a percentage of the optical density values of the control group. The experiments were conducted at least three times by one skilled operator. 

### 4.7. Statistical Analysis

Statistical differences between the experimental groups were determined using the Student’s *t* test. The threshold for statistical significance was set at *p* < 0.05. 

## Figures and Tables

**Table 1:** Primary effects of FGF-2 and PDGF-BB for pulp regeneration.

Growth Factors	Target Cells	Primary Effects
PDGF	dental pulp cells	cell proliferation [14,16,17]
		dentin matrix synthesis [14,16,17]
		odontogenic differentiation
		dentinogenesis [18]
FGF-2	dental pulp stem cells	chemotaxis [19]
		cell proliferation [20]
	dental pulp cells	cell proliferation [21,22,23]
		dentinogenesis [21,22,23]

## Data Availability

Not applicable.
